# Retraction note: Cutaneous mast cell tumor (Mastocytoma): cyto*-*histopathological and haematological investigations

**DOI:** 10.1186/s13000-016-0576-1

**Published:** 2016-11-02

**Authors:** Ehsan Hosseini, Behnam Pedram, Ali Mohammad Bahrami, Mohammad Hossein Jaberi Moghaddam, Javad Javanbakht, Fatemeh Emami Ghomi, Najme Jaberi Moghaddam, Mobin Koohestani, Radmehr Shafiee

**Affiliations:** 1 Faculty of Para Veterinary Medicine, Ilam University, Ilam, Iran; 2 Department of Pathobiology, Susangerd Branch, Islamic Azad University, Susangerd, Iran; 3 Graduate, Faculty of Veterinary Medicine, Urmia University, Urmia, Iran; 4 Department of Pathobiology, Faculty of Veterinary Medicine, Tehran University, Tehran, Iran; 5 Graduate, Faculty of Medicine, Iran University of Medical Sciences, Tehran, Iran; 6 Clinical Biochemistry, Tarbiat Modares University, Tehran, Iran; 7 Graduate, Faculty of Veterinary Medicine, Tehran University, Tehran, Iran

## Retraction

The Editor-in-Chief and Publisher have retracted this article [1] because the scientific integrity of the content cannot be guaranteed. An investigation by the Publisher found it to be one of a group of articles we have identified as showing evidence suggestive of attempts to subvert the peer review and publication system to inappropriately obtain or allocate authorship. This article showed evidence of plagiarism (most notably from the articles cited [2–7]) and peer review and authorship manipulation.
